# Hippo-YAP/TAZ signaling in gastric cancer: orchestrating epithelial-stromal heterogeneity, plasticity, and therapy resistance

**DOI:** 10.47248/chp2603030008

**Published:** 2026-07-02

**Authors:** Junsong Zhao, Curt Balch, Dipti Athavale, Yanting Zhang, Xiaodan Yao, Mikel Ghelfi, Madeline B Torres, Young Ki Hong, Jamin Morrison, Francis Spitz, Generosa Grana, Shumei Song

**Affiliations:** 1. Coriell Institute for Medical Research, 403 Haddon Ave, Camden, NJ 08103, USA; 2. Camden Cancer Research Center, 403 Haddon Ave, Camden, NJ 08103, USA; 3. Departments of Biomedical Sciences, Cooper Medical School of Rowan University, 401 Broadway, Camden, NJ 08103, USA; 4. MD Anderson Cancer Center at Cooper, Cooper University Hospital, 2 Cooper Plaza, Camden, NJ 08103, USA; 5. Department of Surgery, Cooper University Hospital, 3 Cooper Plaza, Camden, NJ 08103, USA

**Keywords:** YAP, TAZ, TEAD1-4, gastric cancer, tumor heterogeneity, cancer-associated fibroblasts, tumor microenvironment

## Abstract

Gastric cancer is a lethal malignancy with highly variable clinical behavior, reflecting both inter-tumor diversity and marked heterogeneity within individual lesions. Large-scale genomics data (including TCGA and ACRG) has delineated recurring molecular subtypes, while expression- and immunohistochemistry-based schemes provide clinically practical surrogates. However, single-cell and spatial profiling reveal that most tumors comprise multiple malignant epithelial states alongside diverse cancer-associated fibroblast (CAF) and immune programs that vary spatially, evolve during progression, and are remodeled by therapy. The Hippo pathway effectors YAP and TAZ act as central integrators of oncogenic, inflammatory, and mechanical cues that coordinate these dynamic states. In gastric cancer, YAP/TAZ activity is enriched in diffuse/EMT-like and mesenchymal phenotypes, associates with peritoneal dissemination, and contributes to immune evasion and treatment resistance. Here, we link YAP/TAZ activity to gastric cancer subtype heterogeneity and emphasize two interconnected dimensions: (i) cell heterogeneity, encompassing coexisting epithelial lineage states and CAF subsets within tumors; and (ii) cell plasticity, highlighting reversible transitions that enable invasion, metastasis, and drug resistance. Finally, we propose a framework of YAP/TAZ-governed epithelial-stromal ecotypes and discuss implications for biomarkers and rational combination strategies incorporating Hippo-targeted agents with chemotherapy, stromal modulation, and immunotherapy.

## 1. Introduction

Despite advances in surgery, chemotherapy, targeted approaches, and immune checkpoint blockade, outcomes for advanced gastric cancer remain poor [1]. A major barrier is heterogeneity across scales: stable genomic and histopathological features coexist with dynamic, non-genetic cell-state diversity in malignant and stromal compartments, generating variable routes of invasion, metastatic tropism, and variable therapeutic responses within and between patients [2-4].

To date, genomic and transcriptomic cluster-based subtype classification frameworks have guided clinical practice [5]. These schemes, however, largely reflect average tumor composition and typically do not capture region-specific lineage mixtures, tumor-stroma architecture, or state shifts driven by inflammation, matrix remodeling, or treatment [6-8]. In contrast, scRNA-seq, spatial transcriptomics, and multiplex imaging show that gastric cancers often contain coexisting malignant states (for example, gastric, intestinal/metaplastic, progenitor-like, and partial EMT programs) [9,10], alongside diverse cancer-associated fibroblast (CAF) and myeloid and T cell states organized into structured spatial ecologies [11,12].

Specifically, the oncoproteins YAP and TAZ (WWTR1), Hippo-pathway transcriptional co-activators, are emerging as key regulators of these ecologies [13]. By integrating receptor signaling, inflammatory cues, and mechanical stress, YAP/TAZ shape transcriptional programs governing proliferation, survival, differentiation, motility, and extracellular matrix (ECM) remodeling, in both tumor cells and CAFs [14,15]. Accordingly, we use YAP/TAZ activity as a unifying lens to link subtype heterogeneity with two intertwined themes: cell heterogeneity (the repertoire of epithelial and stromal states present at a given time) and cell plasticity (their capacity to interconvert during progression and therapy) [16-18].

## 2. Genomic and Cellular Heterogeneity of Gastric Cancer

Classical histopathological systems, such as the Lauren classification, separate gastric cancer into intestinal and diffuse types [19]. Although these categories correlate with anatomical location and clinical behavior, they only partially reflect the molecular diversity revealed by sequencing and transcriptional profiling [20,21].

**Figure 1.**
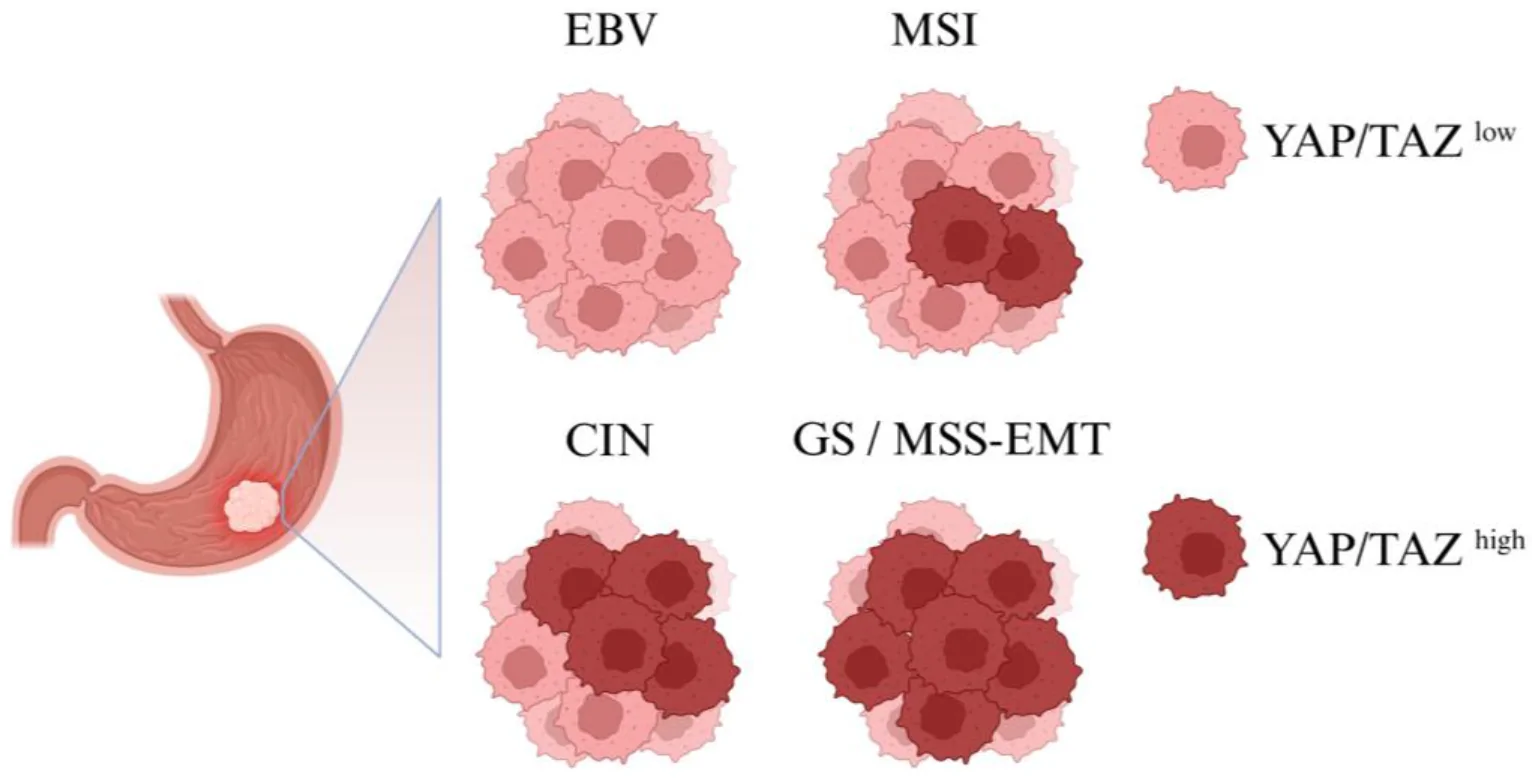
Genomic and cellular heterogeneity of gastric cancer in relation to YAP/TAZ activity. Bulk genomic subtypes (EBV, MSI, CIN, GS/MSS-EMT) show distinct YAP/TAZ activity patterns. Within individual tumors, heterogeneous epithelial lineages and progenitor/EMT-like states coexist alongside diverse stromal programs. YAP/TAZ^high^ progenitor-like and EMT-like cells are most populous in GS/MSS and CIN tumors, as well as in CAF-enriched mesenchymal ecotypes, underpinning tumor plasticity, peritoneal dissemination, and therapy resistance, while less aggressive EBV- and MSI-associated tumors predominantly possess YAP/TAZ^low^ cells. Created with BioRender.com.

The Cancer Genome Atlas (TCGA) has defined several GC tumor subtypes, including Epstein-Barr virus (EBV)-positive (showing PIK3CA mutations and PD-L1 amplifications), microsatellite instable (MSI, showing high mutational burden), chromosome instable (CIN, with recurrent tyrosine kinase amplifications), and genomically stable (GS) tumors, enriched for diffuse histology, which frequently carry alterations affecting adhesion and cytoskeletal signaling. Likewise, the Asian Cancer Research Group (ACRG) reported MSI, MSS/TP53-active, MSS/TP53-inactive, and MSS/EMT-like groups, with distinct recurrence patterns. Among these subtypes, YAP/TAZ^high^ cells are more populous in progenitor-like, EMT-like, and GS tumors with CAF-rich mesenchymal subtypes. In contrast, YAP/TAZ^low^ cells predominate in less aggressive (with inflamed immune programs) EBV-positive and MSI tumors (Figure 1) [22,23], although focal YAP/TAZ niches may still arise at invasive fronts [24,25]. Large-scale analyses further suggest additionally conserved genomic subtypes that refine these frameworks and improve cross-cohort reproducibility [26].

Expression- and IHC-based approaches bridge discovery of subtypes and clinical pathology [27] and can reveal mixtures of CIN-like, MSI-like, and EBV-like features, indicating that clinically intestinal disease may include stromal-rich and EMT-leaning regions with elevated YAP/TAZ [28,29]. Integrative simplified workflows combining molecular class with lymph node status and histopathological features further demonstrate how microenvironmental context modifies clinical risk beyond genotype alone [30]. Early gene-expression studies identified intrinsic classes associated with survival and differential chemotherapy benefit, and subsequent work defined molecular subtypes with distinct responses to PI3K inhibition and 5-fluorouracil, emphasizing that subtype assignments can reveal drug sensitivity [31,32]. Moreover, IHC surrogate panels can approximate TCGA-like classes and capture clinically relevant variation within intestinal tumors, while simplified integrative workflows combine limited molecular testing with routine pathology variables [28-30].

Cellular heterogeneity adds a dynamic layer beyond bulk subtyping [12] with specific cellular combinations enriched in defined niches such as invasive fronts, hypoxic regions, and metastatic deposits [13,33]. State composition can also diverge between primary tumors and metastases, particularly in peritoneal dissemination, where malignant lineage diversity and immune-stromal reprogramming are pronounced [34,35]. Together, these findings argue for subtype models that integrate both genotype (e.g., alterations) and spatial-temporal state expression (e.g., the states of where and when) [36].

## 3. Core Features of the Hippo-YAP/TAZ Pathway in Gastric Cancer

In the canonical Hippo pathway, the MST1/2-SAV1 complex phosphorylates and activates LATS1/2 kinases, which in turn phosphorylate YAP and TAZ, promoting their cytoplasmic retention and proteasomal degradation [37,38]. When Hippo signaling is suppressed, unphosphorylated YAP/TAZ accumulate in the nucleus, where they primarily partner with TEAD transcription factors (along with context-dependent partners such as AP-1, β-catenin, and SMADs) to drive transcriptional programs that promote cell growth, survival, migration, and extracellular matrix (ECM) remodeling [39-41].

**Figure 2.**
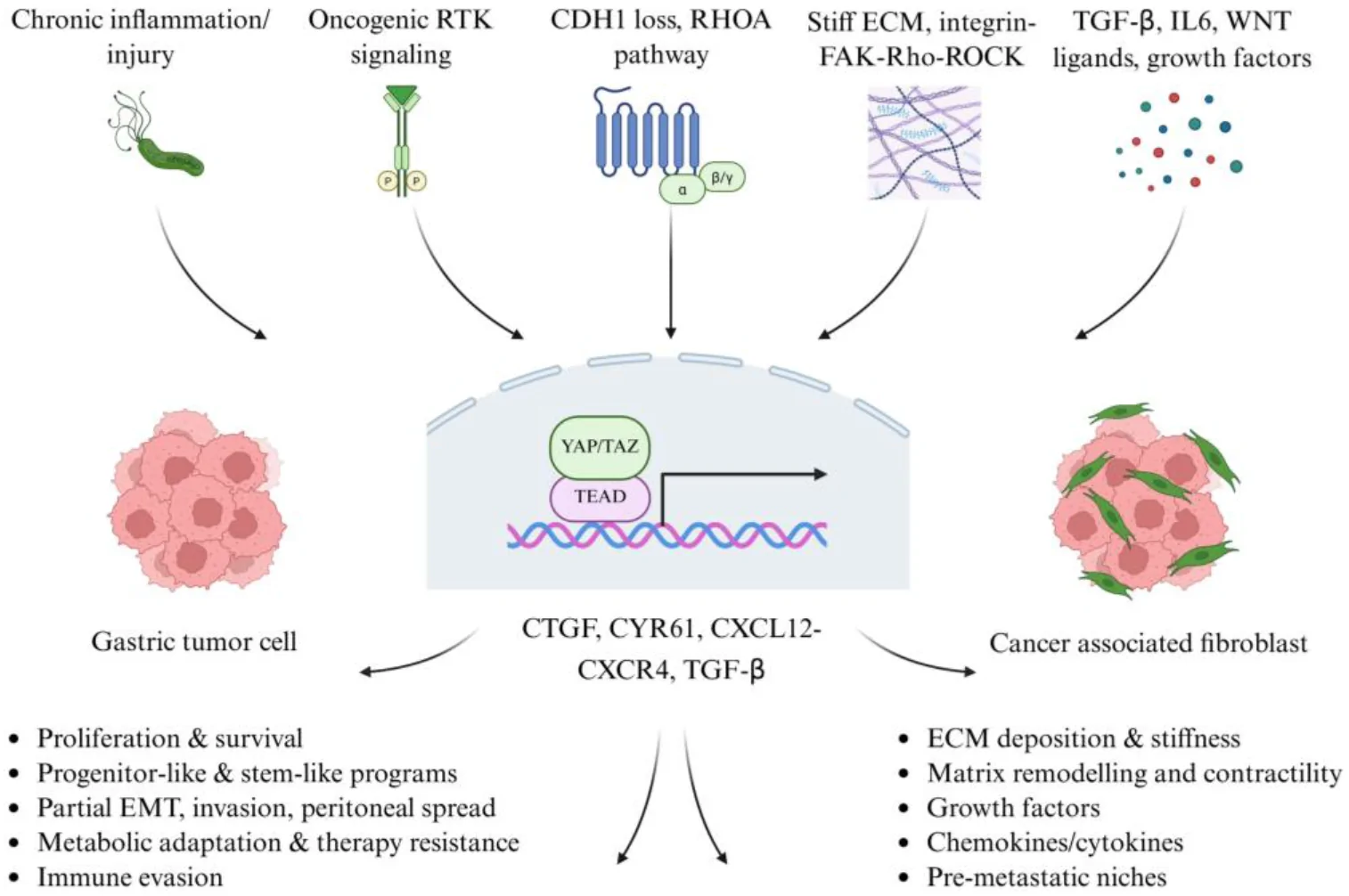
Hippo-YAP/TAZ signaling in gastric tumor cells and CAFs. Multiple biochemical and mechanical cues suppress Hippo kinase activity in gastric cancer, allowing nuclear YAP/TAZ-TEAD accumulation in malignant cells and CAFs. In tumor cells, this drives proliferation, plasticity, invasion, and therapy tolerance; in CAFs, it orchestrates ECM remodeling, fibrosis, paracrine growth signals, and pre-metastatic niche formation. Created with BioRender.com.

As shown in Figure 2, multiple upstream inputs can drive YAP/TAZ activation in gastric cancer [42]. For example, chronic inflammatory cues (including Helicobacter pylori-associated pathways) and growth factor signaling converge on cytoskeletal tension and regulation of Hippo kinase [14]. Genetic lesions enriched in diffuse/GS disease, such as CDH1 loss and alterations in the RHO-family pathway, disrupt cell-cell adhesion and polarity, thereby promoting nuclear accumulation of YAP/TAZ [15,43]. In parallel, receptor tyrosine kinase amplification and integrin-mediated mechanotransduction can sustain YAP/TAZ activity in CIN-like tumors, particularly within stiff, desmoplastic microenvironments [16,17].

Post-translational mechanisms can further fine-tune Hippo signaling pathway activity [44]. For example, ubiquitin-dependent regulation (including deubiquitinases and E3 ligases) controls YAP/TAZ abundance and the stability of Hippo components, creating druggable opportunities to reactivate Hippo signaling (*i.e*., suppress YAP/TAZ activity) or dampen TEAD-driven transcription [20,21]. Because many upstream cues are shared between tumor cells and CAFs, YAP/TAZ can coordinate epithelial and stromal responses to common inflammatory and mechanical stimuli [45].

## 4. Tumor Cell Heterogeneity and YAP/TAZ-Driven Plasticity

Single-cell analyses further highlight tumor diversity [12]. In metastatic peritoneal metastases, malignant cells can show striking lineage diversity, including coexisting gastric and intestinal-like programs within the same patient sample, contributing to intratumoral transcriptomic heterogeneity that is negatively associated with survival [35,46]. Multiplex profiling of peritoneal metastases likewise reveals molecular subtypes and actionable targets that can be obscured by bulk-level averaging [34].

YAP/TAZ play critical roles in maintaining stem-like traits and migratory competence [47]. Mechanistically, YAP can reinforce plasticity through cooperation with lineage and stemness factors; for example, YAP1-induced SOX9 expression promotes stem-like properties in upper gastrointestinal epithelial cancers, illustrating how YAP can couple mechanical or inflammatory cues to lineage regulators in gastric cancer [47-49]. In diffuse-type models, CDH1 loss drives epigenetic reprogramming and immune evasion, changes that may sustain protumorigenic YAP/TAZ activation by weakening junctional restraint and reshaping chromatin accessibility [43,50]. To demonstrate YAP/TAZ GC causation, it was shown that *Lats1/2* knockout (activating YAP1) in pyloric stem cells resulted in proliferation of Lgr5-positive gastric epithelial stem cells, inducing (via Myc) tumorigenic progression from low-grade intraepithelial neoplasia to intramucosal GC [51].

Plasticity becomes most evident during dissemination and under therapeutic pressure [52]. Peritoneal metastasis, common in diffuse/EMT-like disease, causes substantial morbidity and remains difficult to treat [53]. YAP1 is a functional driver of peritoneal metastasis in gastric adenocarcinoma, and YAP1 inhibition can reduce dissemination in preclinical models [48]. To study this, patient-derived peritoneal carcinomatosis cell lines and orthotopic models have now recapitulated key features of human disease, enabling mechanistic interrogation of YAP/TAZ-regulated drug resistance and metastatic behavior [49,54].

Therapy can select for, or actively induce, YAP/TAZ-high tolerant states [55]. Across multiple cancer types, YAP/TAZ signaling is linked to partial EMT, enhanced stress tolerance, and resistance to both targeted therapies and chemotherapy [50,56,57]. As YAP/TAZ activity can promote immune exclusion and dampen antigen presentation through stromal remodeling and cytokine circuits, integrating YAP/TAZ status with immune biomarkers may improve patient stratification [58].

## 5. Cancer-Associated Fibroblast (CAF) Heterogeneity and Plasticity in Gastric Cancer

**Figure 3.**
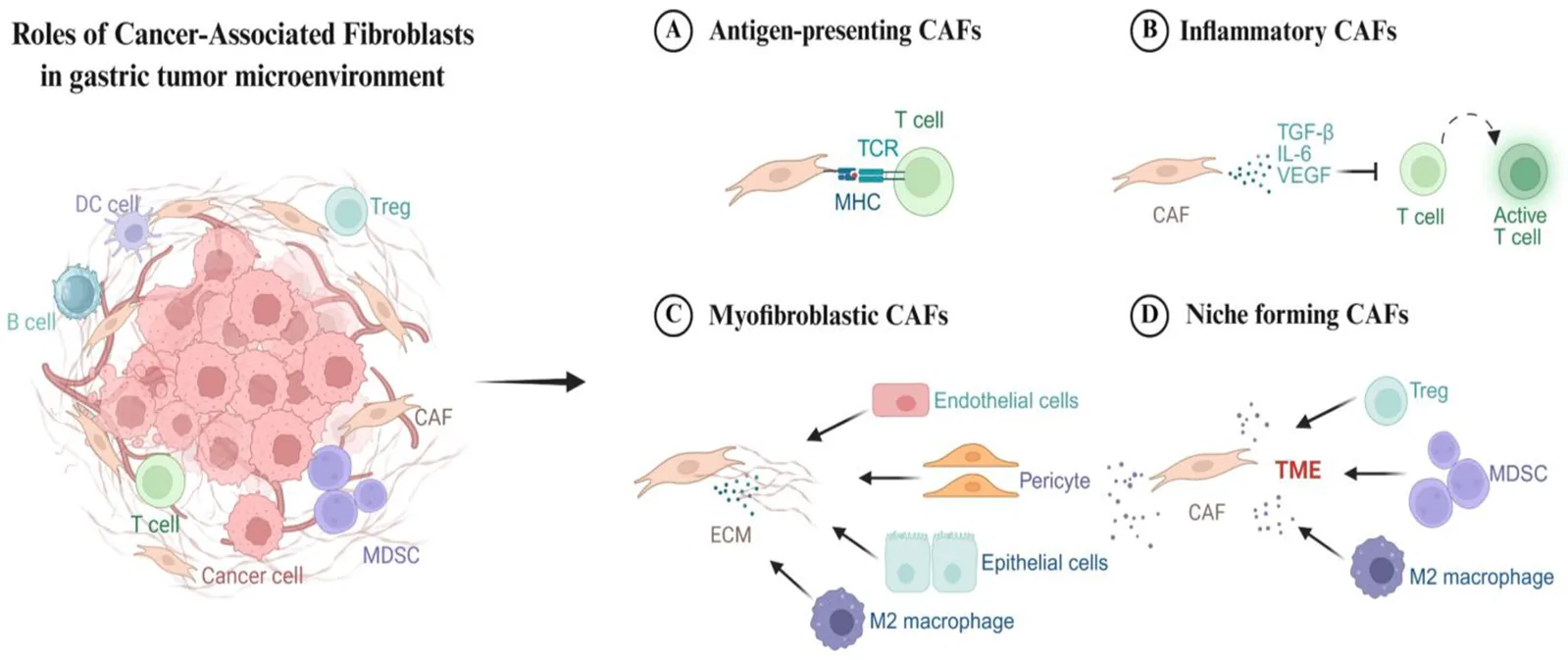
CAF heterogeneity and functions in gastric cancer. Gastric tumors contain myofibroblastic, inflammatory, antigen-presenting, and niche-forming CAF subsets that interconvert under YAP/TAZ control. These CAF programs coordinate ECM remodeling, immune modulation, and pre-metastatic niche formation, promoting tumor growth, metastasis, immunosuppression, and resistance to therapy. Created with BioRender.com.

CAFs are major architects of the gastric tumor microenvironment and are highly heterogeneous [36]. Single-cell and spatial studies have identified distinct subtypes, including myofibroblastic CAFs (myCAFs) with contractile and extracellular matrix (ECM) remodeling, inflammatory CAFs (iCAFs) that secrete cytokines and chemokines, antigen-presenting CAFs (apCAFs) expressing MHC class II molecules, and additional niche-associated fibroblast states that vary across tumors and anatomical sites [59-61] (Figure 3). CAF composition correlates with epithelial phenotype and outcome, with stromal-rich, immune-excluded configurations frequently accompanying diffuse/EMT-like disease [62,63].

CAF plasticity reflects both diverse origins and context-dependent reprogramming [64]. Resident fibroblasts, pericytes, and mesothelial- or epithelial-derived cells can contribute to CAF pools, and exposure to TGF-beta, IL-1, IL-6, and growth factors can shift CAFs between inflammatory and myofibroblastic phenotypes [65,66]. Temporal profiling in other cancers shows that CAF state frequencies change during tumor growth and with therapy, supporting a view of CAFs as dynamic states rather than fixed lineages [67].

YAP and TAZ are central mediators of mechanosensitive CAF activation [68]. Increased ECM stiffness, integrin signaling, and actomyosin contractility drive nuclear YAP/TAZ translocation in fibroblasts, promoting collagen deposition, fibronectin organization, contractility, and secretion of profibrotic factors that further stiffen the matrix [69-71]. This feed-forward loop can both facilitate invasion and impair drug delivery [72].

Distinct YAP/TAZ-dependent CAF reprogramming can also build metastasis-supporting niches [73]. PDPN-positive, LTBP1-positive CAFs promote liver pre-metastatic niche formation through a PDPN-YAP-LTBP1 axis coupled to chemokine-driven recruitment of CCR3-positive myeloid cells, illustrating how specific CAF states can be mechanistically defined and therapeutically targeted [62,71]. More broadly, CAFs reshape tumor ecosystems by modulating ECM biomechanics, growth factor availability, and immune-cell trafficking, implying that CAF-directed therapies should reprogram or selectively disrupt tumor-promoting states, rather than indiscriminately ablating fibroblasts [74,75].

CAF-immune interactions also influence YAP/TAZ-high ecotypes [76]. In gastric adenocarcinoma, tumor-associated macrophage infiltration correlates with PD-L1 expression, linking myeloid context to immune checkpoint signaling within microenvironmental programs that often co-occur with desmoplasia [77,78]. Because YAP/TAZ activity can be amplified by cytokines and mechanical cues generated in CAF- and myeloid-rich niches, myeloid and CAF plasticity should be jointly considered when interpreting YAP/TAZ activity in patient samples and designing combination therapies [79].

## 6. Tumor-Stroma Crosstalk and YAP/TAZ-Defined Ecotypes

**Figure 4.**
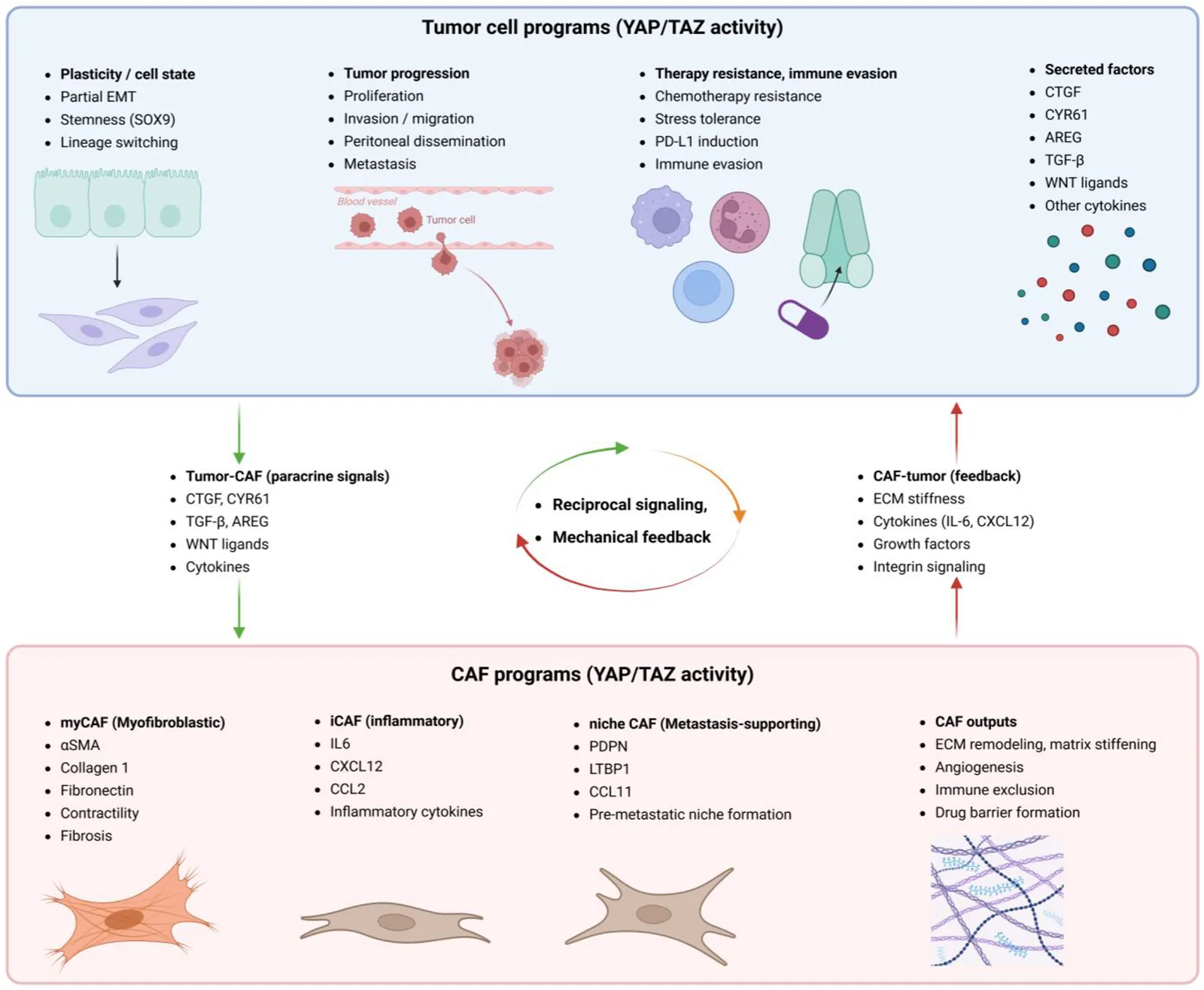
YAP/TAZ signaling orchestrates the crosstalk between gastric cancer cells and cancer-associated fibroblasts (CAFs). YAP/TAZ-driven reciprocal signaling circuit coordinates the crosstalk between tumor cells and cancer-associated fibroblasts (CAFs) to promote tumor progression and therapy resistance. Tumor-intrinsic YAP/TAZ activity fosters cellular plasticity, metastasis, and the secretion of paracrine factors that activate various CAF programs, including myofibroblastic (myCAF), inflammatory (iCAF), and metastasis-supporting (niche CAF) subtypes. These CAF populations, in turn, provide mechanical and biochemical feedback through extracellular matrix remodeling and cytokine secretion, establishing a self- sustaining loop that enhances immune evasion and creates physical barriers to drug delivery. Created with BioRender.com.

Activation of YAP/TAZ programs in tumor cells and CAFs is interconnected through paracrine signaling and mechanical feedback [80]. YAP/TAZ-high epithelial tumor cells can secrete CTGF, CYR61, amphiregulin, and WNT/TGF-β-related factors that activate fibroblasts and promote matrix remodeling. In turn, activated CAFs increase stiffness and release cytokines and growth factors that sustain YAP/TAZ activity in tumor cells, reinforcing malignant state transitions and cellular plasticity (Figure 4) [6,81,82]. This reciprocity suggests that gastric tumors can adopt recurring epithelial-stromal ecotypes characterized by coupled cell-state composition, spatial architecture, and immune context [36]. Spatial and longitudinal profiling across progression supports this model, revealing coordinated evolution of immune and stromal states as tumor-intrinsic and microenvironmental programs co-emerge and co-adapt over time [58].

A clinically important YAP/TAZ-high, EMT/mesenchymal-like ecotype is characterized by hybrid EMT malignant states, abundant contractile CAFs, a dense fibrotic matrix, relative immune exclusion, and a high propensity for peritoneal and liver metastasis [18,19]. Conversely, MSI- and EBV-positive tumors more often display inflamed ecotypes with lower stromal activation, stronger cytotoxic T cell signatures, and a higher response to PD-1 blockade [13,25]. Notably, ecotype switching can occur during therapy and metastatic spread [1].

Conceptualizing ecotypes as dynamic states reframes biomarker priorities: beyond genotype alone, assessments should incorporate YAP/TAZ activity, CAF composition, matrix organization, and immune context in a spatially resolved manner [33]. Serial sampling and non-invasive biomarkers that monitor these features could enable adaptive strategies aimed at constraining and preventing progression towards YAP/TAZ-high tolerant ecotypes [46]. While several such GC ecotypes remain minimally feasible for human clinical use, we note that a machine learning framework, Ecotype [83], has been used to delineate spatial TME profiles in breast cancer [84] and specific, prognostically valuable immunophenotypes in melanoma [85]. Application of such tools to YAP/TAZ GC ecotypes could facilitate their clinical translation.

## 7. Therapeutic Implications

**Figure 5.**
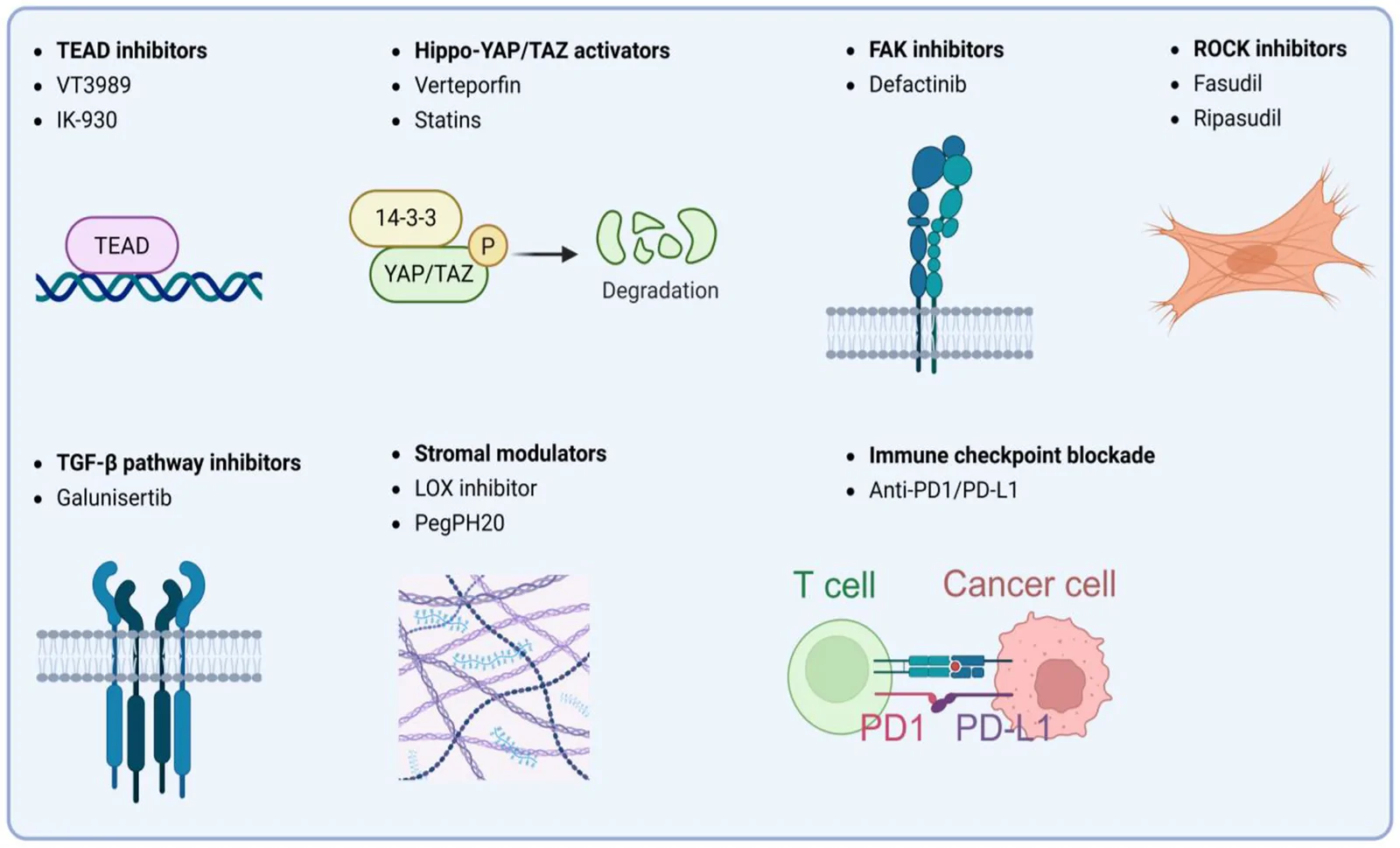
Potential therapeutic strategies targeting the YAP/TAZ signaling network and tumor microenvironment. Various pharmacological strategies aimed at inhibiting pro-tumorigenic signaling and remodeling the tumor niche. These include direct TEAD inhibitors (e.g., VT3989), Hippo-YAP/TAZ activators (e.g., statins) that promote YAP/TAZ degradation, and inhibitors of mechanotransduction (FAK and ROCK inhibitors). Additionally, the schematic highlights the use of stromal modulators (LOX inhibitors, PegPH20) to target the ECM, TGF-β pathway inhibitors (Galunisertib), and immune checkpoint blockade (anti-PD1/PD-L1) to disrupt immunosuppressive loops and enhance therapeutic efficacy. Created with Biorender.com.

Hippo/YAP1-targeting therapeutics remain clinically limited in gastric cancer. However, an ongoing clinical trial (NCT06944249) is examining the effects of a potent YAP inhibitor, verteporfin [86], on fibrosis in surgical wounds, in addition to a phase I (NCT05873686) dose escalation study, in various solid tumors, of the Src family kinase inhibitor NXP900 (which also targets YAP/TAZ signaling [87]). Furthermore, a novel YAP/TEAD inhibitor, VT3989, developed by Vivace Therapeutics, is currently in a clinical trial (NCT04665206) for advanced solid tumors, showing antitumor activity and being well tolerated [88]. Consistent with these clinical efforts, preclinical studies have shown that verteporfin inhibits YAP/TEAD signaling and suppresses the growth of YAP-driven tumors [89,90], while the YAP/TEAD inhibitor CA3 suppresses YAP-dependent transcription, inhibits tumor growth, and enhances therapeutic responses across multiple cancer types [91-94]. A recent study from our group of a novel YAP/TEAD inhibitor, VT00278, that highly suppresses tumor growth by targeting YAP/TEAD signaling and disturbing RNA Pol II activity, enhanced immunotherapy response via an activated cytosolic DNA-sensing pathway in gastroesophageal cancer (Zhang Y *et al.*, MCT 2026 in press). Similarly, the TEAD inhibitors GNE-7883 and K-975 effectively block YAP/TAZ–TEAD signaling and suppress the growth of YAP-dependent tumors [95,96] (Table 1). Such studies could, at a minimum, provide proof-of-concept of YAP/TEAD targeting, including toxicity to normal tissues. In a meta-analysis of 68 solid tumor studies with 8631 patients, tumor YAP1 overexpression negatively affected overall survival, disease-free survival, and recurrence-free survival [97], while in human head/neck squamous cell cancer, YAP-active tumors conferred the worst prognosis of several tumor classification groups [98]. As shown in Figure 5, numerous targeting approaches in gastric cancer are now feasible, including targeting YAP1/TEAD-mediated ECM-stromal-tumor interactions, pathway-focused strategies (FAKi, ROCKi, TGFβi and LOXi, *etc*.), and immunotherapy , in addition to conventional chemotherapies [36]. The therapeutic relevance of YAP/TAZ stems from their position at the intersection of oncogenic signaling, inflammation, and tissue mechanics [59]. Although direct targeting is challenging because YAP/TAZ are non-enzymatic co-activators, several approaches are advancing, including agents that disrupt TEAD palmitoylation or YAP/TAZ-TEAD binding, and strategies that restore Hippo kinase (MST1/2 or LATS1/2) activity [21,42,99]. More advanced strategies targeting YAP/TAZ/TEAD degradation, such as molecular glue and RIPTAC, are now under development. Moreover, YAP inhibition can sensitize YAP/TAZ-dominant cells to cytotoxic therapy. Until completion of clinical studies, however, it remains unknown how YAP targeting would interact with standard-of-care regimens (e.g., HER2-targeted therapies).

In gastric cancer models, pharmacological reactivation of Hippo signaling suppresses YAP activity and increases sensitivity to DNA-damaging chemotherapy, and YAP inhibition can similarly enhance responses in YAP/TAZ-dominant contexts [17,52]. Our previous reports also demonstrated that targeting YAP/TAZ/TEAD can sensitize chemotherapy and radiation therapy [57,100]. These findings support combining Hippo/TEAD targeting with standard cytotoxic regimens, particularly for diffuse/EMT-like and peritoneal metastatic disease, where YAP/TAZ activity is often highest [24,48]. In addition to preclinical rodent models, our group has used patient-derived xenograft (PDX) and patient-derived orthotopic (PDO) models, and lentiCRISPR knockout, to demonstrate YAP1’s critical role in mediating GC peritoneal metastasis [48].

cause mechanical cues reinforce YAP/TAZ signaling, targeting upstream mechanotransduction pathways (for example, focal adhesion kinase, Rho-ROCK signaling, integrin pathways, or collagen crosslinking) may reduce nuclear YAP/TAZ, soften desmoplastic stroma, and improve drug penetration [37,38]. Similarly, selectively disrupting tumor-promoting CAF states (e.g., PDPN-YAP-LTBP1 niche CAFs) or blocking CAF-derived pathways such as TGF-beta or CXCL12-CXCR4 could break epithelial-stromal feedback loops and curb plasticity [62,63].

Immunotherapy adds an additional rationale for combination strategies [25]. Profiling of PD-1 blockade responses in metastatic gastric cancer underscores the need to integrate tumor-intrinsic and microenvironmental determinants [39]. In YAP/TAZ-high ecotypes marked by desmoplasia and immune exclusion, pairing Hippo/TEAD inhibition or stroma-modulating approaches with checkpoint blockade is a logical avenue to test, provided on-target effects on normal tissue repair can be managed through dosing and patient selection [13,40].

Biomarkers should capture both tumor heterogeneity and cellular plasticity [33]. Candidate indicators include nuclear YAP/TAZ localization in tumor cells and CAFs, YAP/TAZ target-gene signatures, ECM/CAF markers (such as PDPN, LTBP1, FAP, and CTHRC1), and spatial metrics of invasive-front ecotypes [71]. Prospective, biomarker-stratified trials will be essential to test whether limiting YAP/TAZ-driven state switching improves durable disease control [41].

Successful therapeutic targeting of the YAP/TAZ signaling pathway still faces many clinical barriers. These include (but are not limited to) intratumoral heterogeneity in YAP/TAZ activity, sampling bias in biopsies, the need for serial or spatial profiling, and the lack of validated, non-invasive biomarkers. Regarding intratumoral heterogeneity (which could be present in biopsies), we note that combination therapies (e.g., chemotherapy plus YAP/TAZ inhibitors) could successfully target low-YAP/TAZ subpopulations. Likewise, spatial profiling technologies are now advancing toward clinical practice by providing "real-time" visualizations of the tumor microenvironment [101]. While noninvasive YAP/TAZ biomarkers remain elusive, studies of specific extracellular oncometabolites or chemokines/cytokines could prove informative in this regard.

## 8. Outlook

Recent genomic, single-cell sequencing, and spatial transcriptomics studies converge on a central conclusion: gastric cancer cannot be fully described by a set of static subtypes [12]. Instead, tumors move through a landscape of epithelial lineages and stromal reprogramming shaped by inflammation, mechanics, and therapy [13].

Framing YAP/TAZ as coordinators of epithelial-stromal plasticity connects genotype, histology, and microenvironmental architecture, motivating new classification and treatment paradigms [33]. Priorities include mapping YAP/TAZ activity and ecotypes in large, clinically annotated cohorts; defining how events such as CDH1 loss and RHOA pathway alterations rewire Hippo dependence; and developing preclinical models that preserve tumor-stroma coupling to evaluate how Hippo-targeted interventions reshape heterogeneity and plasticity [43,49,58].

Ultimately, clinically useful frameworks will need to combine stable molecular strata with dynamic ecotype readouts, enabling therapies that not only match baseline subtype but also anticipate and prevent transitions into drug-tolerant, metastatic states [34,46]. Such approaches could illuminate new means to manage the devastating disease of advanced gastric cancer [35].

## Figures and Tables

**Table 1.** Phenotypic effects and potential mechanisms of YAP1/TEAD inhibitors

YAP/TEAD Inhibitors	Phenotypic Effect	Potential Mechanism(s)	Reference(s)
CA3	anti-tumorigenic anti-proliferation	downregulates YAP1, decreases TEAD transcriptional activity	[91-94,101]
GNE-7883	anti-tumorigenic anti-proliferation	reduces chromatin accessibility at TEAD motifs	[96]
K-975	anti-tumorigenic anti-proliferation	disrupts TEAD-to-YAP interaction	[95]
VT3989	pro-survival clinical tumor responses in a clinical trial (NCT04665206)	blocks TEAD palmitoylation	[88]
VT00278	anti-tumorigenicity anti-proliferation proapoptotic	Suppress YAP/TEAD and disturbs RNA Pol II activity and enhances immunotherapy response via activated cytosolic DNA leading to cGAS-STING activation	Zhang Y *et al*., MCT 2026 in press
verteporfin	anti-proliferation suppresses angiogenesis proapoptotic	sequesters YAP in the cytoplasm by 14-3-3σ	[86,89,90]
